# Progress in Fevers

**Published:** 1903-01-24

**Authors:** 


(*To be continued.*)

### PROGRESS IN FEVERS.

(*Continued from page* 267)

**Typhoid Fever** (*cont.*).—Several cases of *paratyphoid fever* are reported by American observers. W. B. Johnston[8] republishes a case observed by Gwyn, in 1897, in which a "paracolon" bacillus was found. He also publishes notes of four cases recently observed by himself, in two of which an actively motile bacillus was grown from blood cultures. The bacillus behaved in most respects like B. typhosus, but in one case, at any rate, followed B. coli in evolving gas from glucose media. Parallel cultures of Gwyn's "paracolon" bacillus proved the three to be identical. The sera failed to agglutinate B. typhosus, but agglutinative reactions were obtained with organisms derived from other cases of paratyphoid fever. Twenty-six instances of paratyphoid fever are collected, including cases from Paris, Germany, and the Philippine Islands. Paratyphoid bacilli are distinct from B. typhosus, B. coli, and B. dysenteriæ, whilst the work of Widal and others show that they are not identical with the large group of organisms of which B. enteritidis, B. psittacosis, and the bacillus of hog cholera are examples. A gradual onset was generally observed with headache, malaise, loss of appetite and strength, slight bronchitis, and sometimes abdominal pain and chilly feelings. Diarrhœa, rose spots, and enlarged spleen were frequently observed. Clinically the picture is that of a mild typhoid, but two fatal cases are recorded. The disease appears to be rare, for every instance of absence of Widal's reaction is not due to infection with paratyphoid bacilli.

A. W. Hewlett[9] also reviews the literature of the subject, giving details of the behaviour of the bacilli found in some of the reported cases. He thinks the five cases reported by Kurth under the name of Bremen gastric fever may be due to a different organism from that isolated from the cases of Schottmüller and others. A case under his own care showed a well-marked relapse, and all the symptoms of typhoid fever except a palpable spleen and Widal's reaction. A short bacillus was cultivated from the blood which differed from B. typhosus in not producing gas in glucose, and from the colon bacillus in not fermenting lactose, nor coagulating milk, nor readily producing indol. It is pointed out that, as Durham has shown, agglutination reactions, although a very delicate method of differentiating organisms, are not absolutely reliable as tests of the affinity of bacteria. On the one hand closely related organisms may show no mutual serum reaction, and this is especially true of the colon group, whilst, on the other hand, organisms morphologically and culturally distinct may interagglutinate, that is, the serum derived from an animal immunised with the one may agglutinate the other. Cushing's bacillus "O," Gwyn's bacillus, and several others were agglutinated by the serum of a guinea-pig immunised by intraperitoneal injections of Hewlett's bacillus.

Two cases are also reported by W. T. Longcope,[10] one of which proved rapidly fatal, the rectal temperature being 108° immediately before death. No Widal reaction was obtained, but an actively motile bacillus was cultivated from the blood. At the post mortem the spleen was found large, soft, and intensely congested, with some increase in size of the

Malpighian bodies, but neither in the spleen nor in the mesenteric lymph glands, which were of normal size, was any endothelioid proliferation found. No intestinal ulcers were present, nor were any marked microscopical lesions observed. A bacillus was cultivated from the heart's blood, lung, liver, and spleen, morphologically resembling B. typhosus not staining with Gram, but fermenting glucose, dextrose, and mannite. Indol was not formed. From the second case which showed a well-marked relapse, and clinically was indistinguishable from typhoid fever, a similar bacillus was isolated. The serum of this patient gave Widal's reaction with 1 in 20 dilution, so it may have been a case of mixed infection similar to the case reported by Berg and Libman.[11] In this case a well-marked Widal's reaction in dilution of 1 in 200 was obtained, and post-mortem typhoid ulcers were found in the ileum. A paracolon bacillus was obtained during life from the gall bladder, the blood, and the urine.

Sacquépée[12] finds that the serum of typhoid patients frequently agglutinates the colon bacillus, and the same statement is made by others. Burch describes cases of apparently mild typhoid fever not giving Widal's reaction, but yielding growths of bacillus coli communis in cultures made from the urine. Although typhoid fever frequently occurs without intestinal ulceration, absence of all intestinal lesion must be very rare. Opie[13] reviews the literature of this subject. In a case of his own in which Widal's reaction was positive and Eberth's bacillus was obtained from liver, gall bladder, and kidney, death occurred on the twenty-sixth day. Peyer's patches were found unaltered and no hyperplasia or infiltration of lymphoid cells was found on microscopic examination. The mesenteric glands were, however, softened and enlarged, and this fact, together with the presence of abdominal pain and diarrhœa early in the illness, indicate, to Opie's mind, the subsidence of early and slight intestinal lesions. Le Gendre,[14] and also Bezançon and Philibert, on the contrary, report cases of typhoid without gastro-intestinal symptoms. Gall-bladder symptoms were present in one case. Craig and White[15] report a fatal case of continued fever resembling enteric, in which the bacillus enteritidis of Gärtner was found. During life the blood gave Widal's reaction in dilution no higher than 1 in 25. No intestinal ulceration was found post-mortem, nor enlargement of Peyer's patches, only a pretty general bright red injection of the small intestine. The spleen was enlarged and soft, and from it pure cultures were obtained of a motile bacillus morphologically resembling B. coli and B. typhosus. From the former it differed in its cultural characteristics in not fermenting glycerine and in producing pigment as well as gas in formate of soda agar (thrust) cultures, also in not coagulating milk nor producing indol in peptone water. From the latter it differed in producing gas in glucose and formate of soda agar. Its behaviour under cultivation was identical with that of two known strains of B. enteritidis. Disease due to the organisms of the B. enteritidis group, members of which are B. psittacosis and the bacillus of hog cholera, is generally due to eating putrid meat, as in the epidemic reported by Gärtner in 1888, and in several epidemics in this country investigated by Durham and others.

In the treatment of enteric fever Chantemesse[16] is well pleased with the results of his curative antitoxin. Out of 100 cases so treated by him 6 were fatal, of whom three died of perforation, one of pneumonia, and one of a gangrenous bedsore. Injection should be made early in the case, whereupon the temperature and other symptoms subside, almost by crisis, in a few days. The serum should be injected into the forearm, the dose being 10 to 12 cm. No ill effects have been observed from using the serum, which acts, according to Chantemesse, by exciting phagocytosis. Solomon[17] has successfully used arsenite of copper in 27 cases. C. H. Lewis[18] records 90 cases of typhoid fever with six deaths. One or two of the cases were moribund on admission to the hospital. In the majority of these cases calomel and restricted milk with abundance of water were given. Five grains of calomel with 10 of bicarbonate of soda given on admission, and thereafter one grain of calomel in divided doses every 24 hours, with one pint each of milk and of Vichy water and water acidulated with dilute hydrochloric acid, half to one drachm to the quart, "the more the better," was approximately the treatment pursued in most of the cases. D. E. English[19] points out, however, that the average duration of the cases, over 60 days, is very high. A. Koenig[20] also lays stress on the value of abundance of water, from two to three quarts per diem. He administers calomel in doses of one-sixteenth to one-eighth of a grain, and also strongly recommends guiacol. In prescribing the diet, the appetite, and later the tongue of the patient must be considered. As an intestinal antiseptic, A. A. Kent[21] finds 5 grains of sulpho-carbolate of zinc, or 5 grains of thymol useful, in conjunction with some purge as calomel or Epsom salts.

**"Fourth Disease."**—R. W. Marsden[22] reviews the evidence for and against the existence of this disease. In the first place he points out that although Thomas decided that the exanthem of rubeola resembled that of measles only, most writers, including Dukes himself and Watson Williams, describe a morbilliform type and a scarlatiniform type. If "fourth disease" exists, Ker, and apparently also Sir William Broadbent, who considers that "there are at least three kinds of measles," would place it between measles and rubella. Marsden, on the contrary, would prefer to place it between rubeola and scarlet fever. The crucial point in his opinion for establishing Dr. Dukes's claim is whether "fourth disease" does or does not protect from scarlet fever or rubella or measles. F. Caiger does not find that cases answering to "fourth disease" contract scarlet fever in hospital. Scarlatinal rubeola, scarlatinal rubeola and scarlet fever mixed, and mild scarlet fever are the diagnoses which have been made for the three epidemics on which Dr. Dukes bases his findings for a "fourth disease." Whilst far from denying its existence, Marsden demands more conclusive evidence in its favour before accepting the disease as a separate entity.

[8] Amer. Jour. Med. Sci., Aug. [9] Ibid. [10] Ibid. [11] Practitioner, Oct., p. 472. [12] Ibid., pp. 471 and 473. [13] Ibid., p. 470. [14] Ibid, p. 471. [15] Dublin Jour. Med. Sci., Oct [16] Practitioner, Oct., p. 474. [17] Ibid., p. 477. [18] Med. Rec., Aug. 2. [19] Ibid., Aug. 23. [20] Merck's Archives, Aug. [21] Charlotte Med. Jour., June. [22] Lancet, Aug. 16.

*(To be continued.)*

